# Recent Progress on Non-Conventional Microfabricated Probes for the Chronic Recording of Cortical Neural Activity

**DOI:** 10.3390/s19051069

**Published:** 2019-03-02

**Authors:** Chaebin Kim, Joonsoo Jeong, Sung June Kim

**Affiliations:** 1 Department of Electrical and Computer Engineering, Seoul National University, Seoul 08826, Korea; chae.b.kim@gmail.com (C.K.); kimsj@snu.ac.kr (S.J.K.); 2 Department of Biomedical Engineering, School of Medicine, Pusan National University, Yangsan 50612, Korea; 3 Institute on Aging, College of Medicine, Seoul National University, Seoul 08826, Korea

**Keywords:** neural probe, MEMS, chronic recording

## Abstract

Microfabrication technology for cortical interfaces has advanced rapidly over the past few decades for electrophysiological studies and neuroprosthetic devices offering the precise recording and stimulation of neural activity in the cortex. While various cortical microelectrode arrays have been extensively and successfully demonstrated in animal and clinical studies, there remains room for further improvement of the probe structure, materials, and fabrication technology, particularly for high-fidelity recording in chronic implantation. A variety of non-conventional probes featuring unique characteristics in their designs, materials and fabrication methods have been proposed to address the limitations of the conventional standard shank-type (“Utah-” or “Michigan-” type) devices. Such non-conventional probes include multi-sided arrays to avoid shielding and increase recording volumes, mesh- or thread-like arrays for minimized glial scarring and immune response, tube-type or cylindrical probes for three-dimensional (3D) recording and multi-modality, folded arrays for high conformability and 3D recording, self-softening or self-deployable probes for minimized tissue damage and extensions of the recording sites beyond gliosis, nanostructured probes to reduce the immune response, and cone-shaped electrodes for promoting tissue ingrowth and long-term recording stability. Herein, the recent progress with reference to the many different types of non-conventional arrays is reviewed while highlighting the challenges to be addressed and the microfabrication techniques necessary to implement such features.

## 1. Introduction

Microfabrication technologies for neural recording have rapidly advanced over the past few decades in terms of spatial resolution [1,2,3,4,5], topological precision [6], manufacturability [7,8,9,10,11], and multi-functionality [12,13,14,15,16]. Microelectromechanical systems (MEMS) have enabled the fabrication of high-density cortical microelectrode arrays potentially capable of integration with electronics [17,18,19,20,21,22,23,24,25], optic interfaces [26,27,28,29,30,31,32,33,34], and microfluidic channels [35,36,37,38,39,40,41,42,43,44,45], serving as a standard methodology for a wide range of in vivo electrophysiological studies and neural prosthetic devices [46,47,48,49,50,51,52,53,54,55,56,57,58,59,60,61,62,63,64,65,66,67,68,69,70,71,72,73,74,75,76].

**Figure 1:**
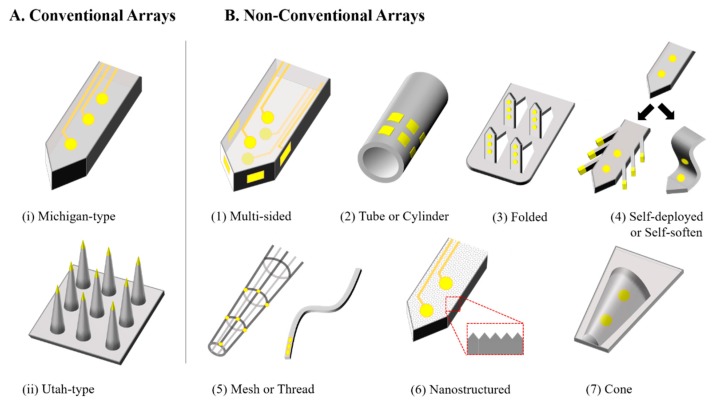
(**A**) Conventional microelectrode arrays including Michigan and Utah probes; (**B**) non-conventional microelectrode arrays with multi-sided, tubular, cylindrical, folded, self-deployable, self-softening, mesh, thread, nanostructured, or conical shape characteristics.

Among a variety of MEMS-based neural probes with diverse geometries, materials, and fabrication techniques, the “Utah” and “Michigan” probe types illustrated in Figure 1B have been most widely used as the standard architecture of microfabricated intracortical arrays [77,78,79,80,81,82,83,84,85,86,87]. Various types of flexible cortical electrode arrays have been demonstrated based on biocompatible polymers such as polyimide, silicone elastomers, parylene or liquid crystal polymers (LCPs) [88,89,90,91]. The integration of additional modalities has gained attention to those seeking to create neural probes with optical stimulation/recording interfaces by patterning wave-guiding structures [92,93,94,95,96,97,98,99] or light-emitting diodes [100,101,102,103,104,105,106] directly onto the shank, or to create probes capable of drug delivery through the formation of microfluidic channels along the probe shaft [107,108,109,110,111,112,113,114,115,116].

While these conventional cortical microelectrode arrays have been extensively used in a number of animal and clinical studies [117,118,119,120,121,122,123,124], there remains room for improvement in the probe structures, fabrication technology and materials, specifically with regard to the chronic implantation of microelectrode arrays [125,126,127,128,129,130,131]. Typical examples of the technical challenges of conventional technology include the shielding of signals from the backside of the arrays, modulus mismatches of devices and the brain, the incapability of accommodating movement of the array within the cortex, immune responses induced by bulky array shanks, and a lack of close integration with neural tissue, all of which contribute to reduced signal quality during chronic recording.

Various research groups have addressed these issues by introducing unique features which are added to conventional shank-type cortical arrays through specially devised microfabrication techniques. As illustrated in Figure 1B, these efforts have resulted in a variety of microelectrode arrays featuring various non-conventional characteristics, including: (1) multi-sided arrays to avoid shielding and increase the recording volume [132,133,134,135,136,137,138,139,140,141]; (2) tube-type or cylindrical probes for three-dimensional (3D) recording, deep insertion and multi-modality capabilities [142,143,144,145,146,147,148,149,150]; (3) folded arrays for high conformability and 3D recording [151,152,153,154]; (4) self-softening or self-deployable probes for minimized tissue damage and an extension of the recording site beyond the gliosis [155,156,157,158,159,160,161,162,163,164,165,166,167,168]; (5) mesh- or thread-like arrays to minimize glial scarring and immune response levels [169,170,171,172,173,174,175,176,177,178,179,180,181]; (6) nanostructured probes to reduce the immune response [182,183,184,185,186,187]; and (7) cone-shaped electrodes to promote tissue ingrowth and long-term recording stability [188,189,190,191,192].

While a number of review papers have thoroughly discussed the design, fabrication, and testing results of conventional microelectrode arrays for the cortex [193,194,195,196,197,198,199,200,201,202,203,204,205,206,207], there are no review articles, to the best of our knowledge, specifically focusing on these types of non-conventional cortical probes containing special geometric features to overcome the various challenges that could not be resolved by conventional arrays. This review, therefore, aims to provide a summarized overview of the various types of non-conventional arrays having unique design characteristics that are differentiated from conventional shank-type probes, covering, e.g., multisided arrays, mesh- and thread-type arrays, cylindrical arrays, folded arrays, self-deployed arrays, nanostructured arrays and cone-shaped arrays. We aim to highlight the challenges to be addressed and the microfabrication techniques required to implement them for each specific structure in the following sub-chapters.

## 2. Non-Conventional Neural Probes

### 2.1. Multi-Sided Probes

Conventional MEMS-based microelectrode arrays typically carry neural interfacing electrodes only on a single side of the probe shaft. This stems from the nature of MEMS technologies, where each conductive or insulating layer is sequentially deposited and patterned on top of a substrate material. Neural signals from the backside of the probe are partially shielded by the probe shank, limiting the volume of tissue being recorded or stimulated to the neurons facing the front of the array. Recently, many research groups have developed multisided cortical arrays in which two or more sides of array shanks are formed with microelectrode sites by introducing special schemes during the microfabrication process. These multi-sided neural probes can provide the advantages of an increased recording volume without a shielding effect as well as an increased number of sites without significantly increasing the probe size under the same line width or spacing.

**Figure 2:**
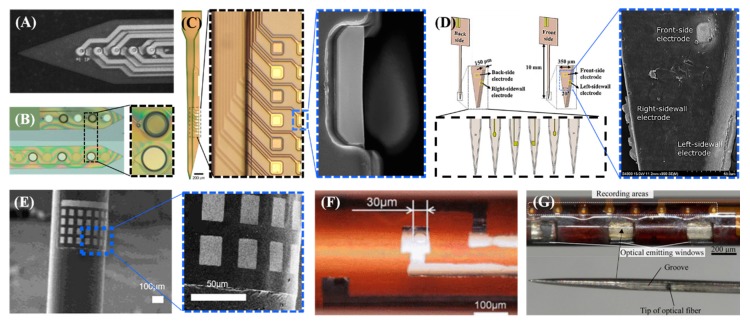
Featured multi-sided probes: (**A**) double-sided, transparent polyimide probe (reprinted from [134] with permission from Elsevier, Copyright 2002,); (**B**) double-sided polyimide probe fabricated using silicon posts for opening the electrode on the first metal layer reprinted from [138], with permission from IEEE, © 2012); (**C**) triple-sided parylene probe (reprinted from [140] with permission from Springer Nature Customer Service Centre GmbH); (**D**) four-sided probe based on a liquid crystal polymer (reprinted from [141] with permission from Elsevier, Copyright 2017,); (**E**) Nanoelectronics coating onto optical fiber using Van der Waals interaction (reprinted from [109] with permission from The American Chemical Society, Copyright 2017); (**F**) parylene/polyimide hybrid probe fabricated by direct patterning on a cylindrical surface (reprinted, from [107] with permission from IEEE, © 2017); (**G**) tube-shaped polyimide probe in which a sharp metal needle and optical fiber are embedded (reproduced from [106] with permission from John Wiley & Sons, copyright 2017).

Dual-sided arrays with sites on both the tops and bottoms of the probes have been proposed by several groups [132,133,134,135,136,137,138,139]. Common challenges when creating dual-sided probes include how to minimize the number of process steps and how to align the top and bottom patterns. The simplest approach is to complete one side first and then turn it over to repeat the same steps on the back [132,133,134,135]. Several alignment methods have been proposed for silicon probes by, for instance, creating through-hole alignment marks [132] or using optically transparent adhesives with a glass carrier [133]. Alignment has not been a problem for arrays based on a transparent polymeric substrate such as polyimide (Figure 2A) [134]. Dual-sided probes can also be created through special single-side processes without flipping the samples by forming the bottom metal layer first and open them at the last step by etching the silicon carrier from the backside [137]. Alternatively one can make silicon posts for the recessed opening structure of the bottom metal layers in the first step, which removes the need for a backside opening step, as in Figure 2B [138], or form glass within a silicon mold and then create top and bottom layers after etching the silicon mold [139].

A three-sided array was developed by Seymour et al. [140]. Electrodes were made on the top, back and one of the edge of the devices by introducing a chemical mechanical polishing process and utilizing a structured SiO_2_ layer as a release layer and a site place holder, as shown in Figure 2C. The side electrode had the unique feature of three exposed sides. A four-sided depth probe was developed by Shin et al., having electrode sites on all four sides of its rectangular probe shank based on liquid crystal polymer (LCP), as shown in Figure 2D [141]. Four metal patterned LCP layers for the top, bottom, and two side electrodes were stacked and thermally laminated together with cover LCP layers. The top and bottom electrodes were created by laser opening windows through the cover LCP layers. Metal lines for two side electrodes were formed to extend beyond the laser-cut outline of the shank such that the cut-away cross-section of the electroplated metal lines (5 μm-thick) were used as the recording surfaces. The thermoplastic properties of LCP were utilized to create a multilayered substrate simply through single-step thermal lamination process enabling high scalability of channel density. While these multisided probes have been verified in animal experiments using rats [133,135,140], mice [141], and guinea pigs [136,137], their use is not limited to small animals but could be applied in large animals or clinical trials in the future studies given the shape of the shank is not very different from the conventional Michigan arrays.

### 2.2. Tubular or Cylindrical Probes

Various types of brain probes with tube- or cylindrical structures have been demonstrated, offering several advantages over conventional shank-type probes. The hollow core of the tube structure can be utilized to add multi-functionality by inserting optic fibers or flowing drugs through the channel without increasing the probe dimension [145,146,147,148,149,150]. This type can also be exploited to control the flexibility of the probe by making it rigid with mechanical support during the insertion process and retuning it to highly flexible state after positioned in the brain and guide removal [146,149]. Moreover, three-dimensional probing and high spatial selectivity can be achieved by placing electrode sites around the circumferential surface of the tube [142,148,150]. Lastly, the cylindrical structure is mechanically favorable for deep brain insertion with a small impaired brain volume, which can be beneficial for the application of deep brain stimulation (DBS), stereoelectroencephalography (SEEG), and neural recording in deep brain regions [142,143,144,148,149].

Fabrication techniques of three-dimensional tubes or cylindrical structures can generally be categorized into three types: creating a conventional planar thin-film array and wrapping it around a shank [142,143,144,150], direct patterning onto a curved cylindrical surface using customized non-planar microfabrication tools [145,146,147,148], or building a three-dimensional tube structure starting from a planar substrate by sequential deposition and etching [149]. A polyimide-based thin-film electrode array fabricated by conventional microfabrication process was wrapped around and fixed onto a polyurethane shaft at a 240-degree angle by thermal forming of a thin film array between a pair of matched molds [142]. Another group utilized a micro-patterned fluoropolymer layer (CYTOP) as a thermally actuated adhesive to retain the cylindrical shape after inserting a rolled electrode film into a custom metal mold containing a cylindrical hole with an entrance slit, followed by thermal annealing and epoxy filling [143,144]. Both abovementioned approaches are aiming at three-dimensional probing for DBS applications. A recent study proposed a “nanoelectronic coating” in which an ultrathin (less than 1 μm) electrode array based on SU-8 was conformally adhered to the curved surface of the host probes by Van der Waals interaction after a special wetting and lifting process, as shown in Figure 2E [150]. The host can be either optic fiber or a micropipette such that multi-functionality is enabled with a negligible change in the volume [150].

While non-planar photolithography techniques for the direct patterning of microstructures onto the curved surfaces of polymer tubes require complicated tooling, this non-planar patterning method can create a tube with a smaller diameter (~300 μm) with high throughput and without a manual rolling process. Typical non-planar microfabrication setups involve a UV laser projection system with a synchronized multiaxial motion stage for uniform exposure throughout the entire surface in addition to a rotation mechanism for metal deposition and photoresist coating, as presented in Figure 2F [145,146,147,148]. A sharp metal needle embedding an optic fiber with a mirror at the tip was inserted into the tube structure to facilitate cortical insertion and to deliver multiple-depth optical stimulation and electrical recording, as shown in Figure 2G [145,147].

A tube structure could also be created by a series of special MEMS processes on a planar substrate by deep silicon etching and parylene deposition to form sidewalls and a ceiling, followed by XeF2 silicon etching to create a hollow space, which could be used for drug delivery or optic fiber insertion [149].

The functionality of these tubular probes haven been demonstrated in animal tests using rats [145,146,147,149], mice [142,150], or monkeys [144], with potential applications for human targeting cortex [142,143,144,148,149] and deep brain regions [142,143,144,148,149].

### 2.3. Folded Probes

Conventionally, three-dimensional recording probes with both lateral and depth-wise access in the cortex could be fabricated typically by the stacking of multiple two-dimensional silicon probes, which requires precise assembly and interconnection schemes [82,83]. These rigid probes cause tissue inflammation during in vivo recording due to the mechanical mismatch between the rigid shank and the soft tissues. 

Flexible polymer-based foldable arrays have been developed to address this issue, in which a non-planar structure could be created by folding some parts of the thin-film array by means of magnetic force [153,154], electrostatic force [151], or origami-like manipulation [152]. These types of folded probes can offer three-dimensional recordings of brain activity in both horizontal and vertical directions, yet with a high degree of flexibility conforming to the curvature or movement of the brain tissue. The capability of integrating both a surface-type and penetrating electrode onto a single device was also presented [152].

**Figure 3:**
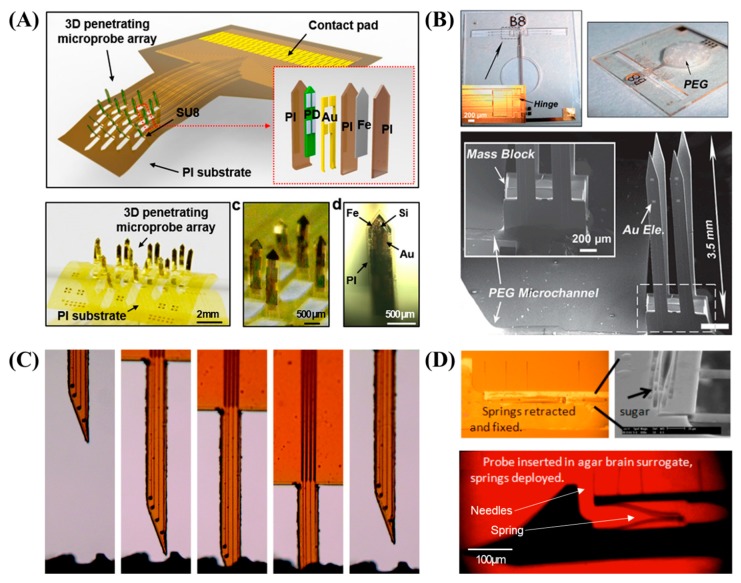
(**A**) Polyimide optrode array folded using magnetic force (reproduced from [153] with permission from Springer Nature, copyright 2018. Creative Commons Attribution 4.0 International License: http://creativecommons.org/licenses/by/4.0/); (**B**) neural probe consisting of a SU-8 shank and a parylene hinge and folded using electrostatic force (reproduced from [151] with permission of The Royal Society of Chemistry); (**C**) probe based on a ternary thiol-epoxy/maleimide network for softening after implantation (reproduced from [159] with permission of The Royal Society of Chemistry); (**D**) silicon neural probe with satellite recording sites that can be self-deployed (reprinted, from [167] with permission from IEEE, © 2011).

Thin layers of micromachined magnetic materials, such as electroplated nickel [154] or evaporated iron [153], were embedded into the penetrating parts of planar polymer-based arrays with the surrounding cut out such that magnetic actuation could force the penetrating parts to be folded out of the plane and up in the vertical direction. In one study [154], six fingers 1.2 mm in length and 160 μm in width were successfully folded at 90 degrees by applying a magnetic field of 380 mT, but this method has a limitation in that it requires the dura to be removed before the insertion into the rat brain due to the lack of rigidity of the structure. Small droplets of SU-8 were placed on the hinges of each bending area of 4 by 4 penetrating probes which were cured while a magnetic field was applied such that the 3D probe configuration was fixed even after removing the magnetic field, as shown in Figure 3A [153]. Electrostatic force was employed as an actuating mechanism in another study [151] to avoid the use of toxic magnetic materials such as nickel and iron, where electrostatic potential at 8 kV could fold four shanks 3.5 mm long and 200 μm wide to a near upright position, as shown in Figure 3B. Here, the penetrating probes were mechanically reinforced by patterned SU-8 layers backing a flexible parylene substrate and with the injection of PEG into the hinge area, which was shown to have sufficient rigidity to penetrate into the brain-mimicking bio-gel [151].

A completely different concept of folding was also proposed when researchers introduced an origami-like method to combine epicortical ECoG and an intracortical depth probe into a single device [152]. The planar polyimide electrode array consists of microfabricated electrodes and a set of cut-outs that allow the penetrating portions to remain ‘in-plane’ while the folded portions form a surface-type ECoG array when manually folded and plugged into the connector, which was tested in rat brain.

### 2.4. Mechanically Dynamic Probes (Self-Softening or Self-Deploying Probes)

One of the major challenges related to the chronic implantation of conventional probes is tissue damage and scar formation around the implanted probe due to the considerable mechanical mismatch between the tissue and the silicon probes, and the forces exerted by the micro-motions of brain tissue after implantation, all of which eventually cause degradation of the signal quality. Flexible thin-film polymeric probes can be used for high conformability to brain tissue, but they are not rigid enough to penetrate into the cortex such that a special delivery tool or stiffening by biodegradable materials is required. Mechanically dynamic probes have been demonstrated to overcome the abovementioned limitations, with some parts of the probes designed to self-soften or self-deploy in response to physiological conditions using thermally sensitive or water-sensitive composite materials [155,156,157,158,159,160], shape-memory polymers [161,162,163], special device layouts [166,167] or built-in stress [168].

Self-softening probes are initially stiff enough to facilitate cortical insertion with minimized trauma, but they become mechanically compliant post-implantation to closely match the modulus of the brain tissue while minimizing mechanical stress and glial reaction. This transition of the mechanical properties triggered by heat or water can be implemented using special composite materials as a probe substrate, including nanocomposites [155,156,157,158,159], off-stoichiometry thiol-ene-epoxy (OSTE+) [164], thiol-ene/acrylate [160], and shape-memory polymers (SMPs) [163]. The extent of modulus changes and the glass transition temperature of these mechanically active materials could be adjusted by tailoring the polymer compositions. In several works [155,156,157], a nanocomposite consisting of a cellulose nanofiber encased in a polyvinyl acetate matrix was utilized to create a rigid array having a Young’s modulus of a few GPa, which could be softened to ~10 MPa in response to water in the implanted environment. SMPs are used to store the metastable shape, which can return to a globally stable shape once activated by environmental triggers such as temperature, heat, humidity, or light [165]. While SMPs have previously been utilized for electrode arrays with three-dimensional structures, such as a cuff-type array, recent work has proposed a cortical array using thermally sensitive and water-sensitive SMPs, the modulus of which is softened from 700 MPa to 300 kPa after implantation [163]. A similar result was obtained using thiol-ene/acrylate, which softens due to the presence of water. OSTE+ is a heat-sensitive epoxy material with a Young’s modulus that changes from more than 1 GPa at 10 °C to 30 MPa at 40 °C [164]. Recently developed composite materials include a polyvinyl acetate matrix with cellulose nanocrystals which can soften from 5 GPa in a dry state to 10 MPa in a wet state [158] and a thiol–epoxy/maleimide system which can soften from 1 GPa in a dry state to 30 MPa in a wet state (Figure 3C) [159].

Some parts or the entire array of self-deployed probes can physically move toward target locations populated by neurons that are more viable and less impaired by the insertion of a probe shank [166,167,168], generally away from the shank beyond the glial scar. A satellite shank with electrode sites connected to the main shank via a micro-spring structure can be created by DRIE of a SOI wafer, which could then be retracted before insertion by capillary force and dissolvable glue. This type of satellite shank was deployed after insertion by releasing the spring to extend the electrode sites toward neurons away from the main shank, as in Figure 3D [167]. Silicon-based compliant whiskers could splay outward, extending beyond the typical 50–100 μm radius of the glial formation, with potential recording site patterning on the tip of the whisker [166]. The built-in stress of thin-film conducting layers of Cr/Pd/Cr can be utilized to bend the tip conductor of the probes off of the substrate to locate the recording site away from a lesion caused by surgery [168]. Insertion-induced tissue damage could be reduced by the extremely slow implantation of a SMP probe. This is initially implanted into the brain in a crouched shape, after which heat from the body triggers slow actuation of the probe back to its original shape, moving the probe tip beyond the initial insertion and toward the target location. No further damage was caused during this step, as the extension speed of the SMP probe (e.g., 120 μm in 1.5 days) is comparable to the rate of cell migration [162]. Some of the abovementioned probes were tested in animals including rat [155,158,163], mice [160], or cockroach [156].

### 2.5. Mesh and Thread Probes

Minimizing the reactive tissue response is the key factor during the chronic recording of neural activity [144,145]. The tissue response is related to blood vessel damage during implantation due to the friction and cutting forces [208,209,210] and micro-motion-induced local stress after implantation [211,212,213]. The use of substrate materials that are as flexible as brain tissue reduces the chronic foreign body response, but current materials for neural probes have elastic modulus values higher by multiple orders relative to those of brain tissue [214]. To overcome this limitation, ultra-thin or ultra-compliant probes have been demonstrated to minimize how much tissue displacement and local stress affect neural tissue by employing thread- or mesh-like ultrathin structures of polymer materials [215]. This category of neural probes includes ultrathin and open mesh structures [169,170,171,172,173,174], ultra-flexible nano-scale thread structures with a subcellular cross-sectional area [175,176], thin shaft structures embedded in a bio-dissolvable needle [177], and sinusoidal structures to reduce the tethering force [178,179,180,181].

**Figure 4:**
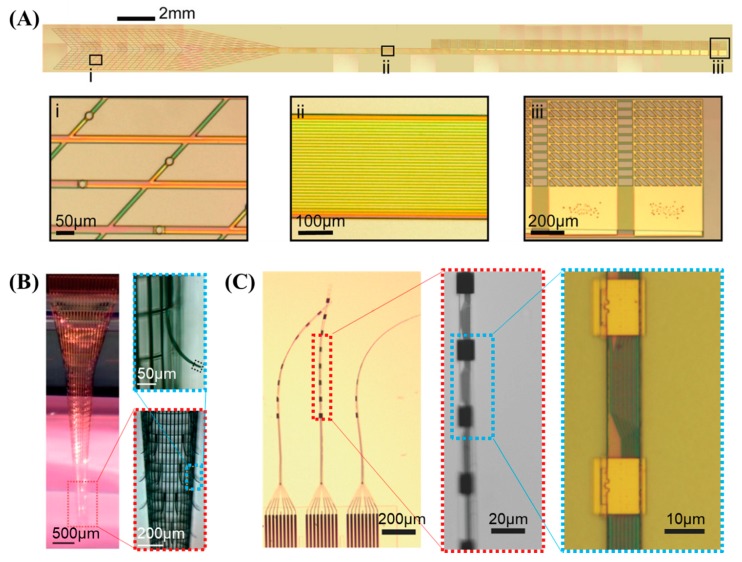
Mesh and thread probes: (**A**) syringe-injectable SU-8 mesh electronics with a plug-and-play input/output interface (reprinted from [173] with permission from American Chemical Society, Copyright 2017); (**B**) cylindrical, self-scrolled SU-8 mesh electronics (Reprinted from [170] by permission from Springer Nature Customer Service Centre GmbH, 2015); (**C**) nanoelectronic thread probe (reproduced from [176] with permission from John Wiley & Sons, copyright 2018).

Highly compliant sub-μm mesh electronics have been demonstrated to be capable of minimizing the stress exerted onto the tissue while also allowing neural cells, nutrients, and cytokines to interpenetrate through the open space of the mesh [174,216]. Acute and chronic tissue responses could be effectively suppressed such that neural activities from identical individual neurons can be recorded over a time period of eight months [216,217]. The ultra-thin mesh structure is composed of SU-8 thin mesh frames 5–20 μm wide with embedded interconnection wires and recording sites configured in a longitudinal/transverse mesh or rhomboid mesh layout having open spaces ranging from 60 to 300 μm, as shown in Figure 4A,B. These probes were fabricated by the direct photolithography and etching of SU-8, while E-beam lithography was employed to increase the channel density to 128 channels and decrease the cross-sectional area of the mesh frames [169]. Insertion into the rat or mouse brains is facilitated by freezing the probe rapidly in liquid nitrogen to enhance its stiffness temporarily [170], which later evolved to “syringe-injectable electronics” in which the mesh electronics section was loaded into a syringe needle and implanted into the target position within the cortex by the tailored coordination of a syringe pump and a motorized stereotaxic stage [171,172]. Injectable electronics were interconnected to external circuitry after implantation by means of anisotropic conductive film [172], conductive ink printing [171], or a conventional zero-insertion-force (ZIF) connector [173].

The nanoelectronic thread (NET) electrode is an SU-8-based ultrathin thread-like single strand with a typical cross-sectional profile of ~ 0.5 × ~ 10 μm^2^ hosting up to 16 recording sites along the shaft, as shown in Figure 4C. The width of the thread can be effectively minimized by placing the interconnections and electrodes on different layers and connecting them by vias through the insulation, which are fabricated by a combination of e-beam lithography and photolithography. The subcellular surgical footprint could reduce the bending stiffness and tissue displacement, thus improving the long-term reliability and glial scar-free neural integration. When applied, it enabled stable cortical recording from a mouse for a period of four months [175,176]. During implantation, the tip of a sub-10 μm microshuttle device made of carbon fiber was fitted into a microhole at the end of a NET probe for delivery into the desired location in the mouse brain.

Other common approaches for reducing glial scarring involve the use of flexible polymer-based probes with minimized cross-sectional areas, temporarily strengthened by biodegradable supporting materials during insertion [178,179,180,181]. A typical substrate material is parylene or polyimide, forming a highly conformable probe shank with a cross-section in the range of 2.7–20 μm by 10–35 μm [177,178,179]. These probes can feature special layout schemes, such as wavy [179,181] or sinusoidal probe shanks to achieve smooth stress distributions throughout the length of the probe [178], and a spheroid tip to minimize tissue damage and serve as an anchor for the recording sites [178]. The insertion could be facilitated by embedding the probe in a biodegradable material such as carboxymethylcellulose [180] or silk [175], which could also be aided by insertion carriers in the form of syringe needles, carbon fiber, and/or tungsten microwire. After dissolution of the support, these compliant probes have been shown to provide stable cortical recording for up to 678 days from rabbits [178].

### 2.6. Nano-Structured Probes

**Figure 5:**
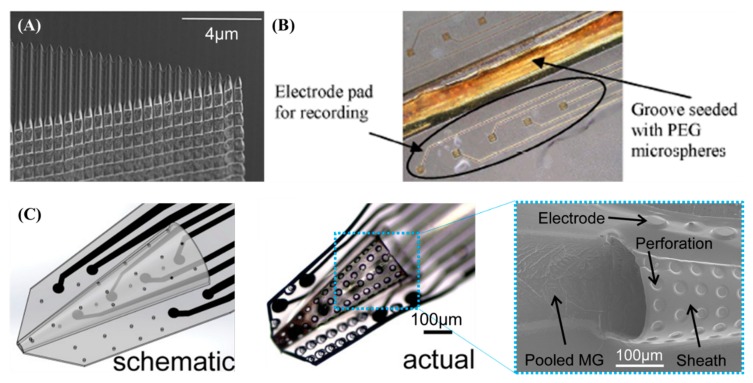
Nanostructured and sheath probes: (**A**) probe having a nano-groove for reduced immune reaction (Reproduced from [186] with permission by John Wiley & Sons, 2017); (**B**) probe having a micro-groove to deliver bioactive molecules (reprinted from [183] with permission from IEEE, © 2006); (**C**) probe having a truncated cone sheath with perforations and a matrigel coating (reproduced from [192] with permission by John Wiley & Sons, 2015).

Cortical probes with microscale or nanoscale surface texturing have been proposed to reduce the immune response and prompt neural integration near recording sites for improved chronic performance [182,183]. Nano-pores [184], nano-pillars [185], and nano-grooves [186] of a few hundred nanometers were formed on the surface of the silicon probes by anodic etching, deep reactive ion etching (DRIE), and focused ion beam etching, respectively (Figure 5A). These nano-textures could mimic the natural extracellular matrix, effectively suppressing gliosis and neuronal loss, as demonstrated by in vitro and in vivo experiments in which negative factors potentially degrading chronic neural recording, such as the growth of astrocytes around the surface, were significantly lowered on the nano-textured probe surfaces in comparison with flat surfaces. Positive factors such as neurite extension or the number of neurons in proximity (< 50 μm) to the surface were meaningfully improved in rat experiments [184,185,186]. Furthermore, the nitric oxide synthase (NOS2) gene expression of a nano-grooved probe was significantly lower than that of a flat probe, indicating potentially less neurotoxicity of the nano-grooved probes [186].

Micron-scale surface structures such as microwells [182,187] and micro-grooves [183] with dimensions of tens to a hundred micrometers have been fabricated typically on polymer-based probes by photolithography and etching. These micro-textures can be utilized to embed bioactive molecules typically by direct seeding into wells [187] or by gluing microspheres containing neural growth factor onto the groove [183] for delivery into the targeted cortical region with spatiotemporal precision (Figure 5B). An in vitro analysis showed extended neurites of neurons, and an in vivo analysis showed an increased protein (laminin) level in the extracellular matrix.

### 2.7. Sheath Probes

Parylene C neural probes with a three-dimensional sheath structure were demonstrated to have a truncated cone at the tip of the probe with a length of 800 μm, a base diameter of 300–450 μm, and a tip diameter of 50 μm. The goal of this was the ingrowth of neural cells and thus enhanced tissue integration around the recording sites lining the interior and exterior regions of the cone sheath [190,191]. The sheath structure was fabricated by creating a microchannel using a sacrificial photoresist followed by the thermoforming of the microchannel with a microwire mold inserted into the channel. Perforation (15 μm diameter) of the sheath structure could be utilized to enhance the transduction of cytokines and nutrients (Figure 5C) [188,189]. For enhanced neural integration, a cocktail of membrane proteins and growth factors (matrigel) was coated onto the lumen and surrounding edges by treating parylene with poly-D-lysine followed by dipping into the mixture [192]. In vivo studies using rats verified stable neural recording for 12 months [189]. The matrigel coating increased the level of neuronal attachment and hence enhanced the quality of the recorded neural signal from rat brains [192].

## 3. Conclusions and Perspectives

Undoubtedly, stable neural recording during the chronic implantation of intracortical microelectrode arrays is of paramount concern for the successful implementation of neural prosthetic devices such as brain-machine interfaces (BMIs) and for a better fundamental understanding of brain functions. Despite the fact that the field of microfabricated cortical probes represented by Utah or Michigan arrays have witnessed remarkable advancements over the past few decades, there remain technical challenges and limitations resulting in degraded or inconsistent recording quality levels and/or electrode failures over time. The approaches introduced in this article are in the form of a summarized overview of the various types of unconventional cortical probes featuring unique design characteristics which specifically overcome the limitations of conventional shank-type probes with regard to chronic cortical recording. These unconventional designs include multi-sided arrays, mesh- or thread-like arrays, tube-type or cylindrical probes, folded arrays, and self-deployable probes, with the common goal of stable and high-quality recording of neuronal activity from the brain for extended periods of time. The new ideas applied to these unconventional probes have been significantly effective to address various targeting issues by minimizing neural damage during and after insertion, adding the potential capability of multi-modality, and increasing the three-dimensional recording volume.

It should be noted, however, that none of the abovementioned technologies can resolve all of the challenges at once. Therefore, major technical breakthroughs are expected in the fields of neural engineering and neuroscience by those who incorporate many existing solutions into a single device, enhancing our understanding of the brain and bringing the practical use of BMI technology far closer to reality. This is why we should be encouraged to continue to pursue new technical developments and exciting solutions to meet the requirements of a high-density and high-fidelity cortical interface for brain science and engineering.

Although this review is focusing on the microfabricated probes, a variety of arrays not fabricated through MEMS technology also comprise an important part in the field of cortical probes. These types of probes are typically based on precise assembly of bundle of wires such as thin metal wires, carbon fibers, carbon nanotube (CNT), and/or optic fibers to create neural interfaces with high-density and multi-modality [218,219,220,221,222,223,224]. Finally, we also note a set of emerging technologies in the field of brain engineering, typically consisting of miniaturized (sub-mm) implants with single-channel recording (and/or stimulation) with wireless power and communication capabilities that can be fully implanted into the brain with a minimized form factor. These devices include those termed as the Neural dust [225,226,227] and Neurograin [228], which differ in terms of their power supply, communication, packaging, and circuit design.
